# Single-Round Infectious Particle Production by DNA-Launched Infectious Clones of Bungowannah Pestivirus

**DOI:** 10.3390/v12080847

**Published:** 2020-08-04

**Authors:** Anja Dalmann, Kerstin Wernike, Eric J. Snijder, Nadia Oreshkova, Ilona Reimann, Martin Beer

**Affiliations:** 1 Institute of Diagnostic Virology, Friedrich-Loeffler-Institut, 17493 Greifswald-Insel Riems, Germany; anja.dalmann@fli.de (A.D.); kerstin.wernike@fli.de (K.W.); 2 Molecular Virology Laboratory, Department of Medical Microbiology, Leiden University Medical Center, 2333 ZA Leiden, The Netherlands; E.J.Snijder@lumc.nl (E.J.S.); nadia.oreshkova@wur.nl (N.O.)

**Keywords:** Bungowannah virus, flavivirus, reverse genetics, single round infectious particle

## Abstract

Reverse genetics systems are powerful tools for functional studies of viral genes or for vaccine development. Here, we established DNA-launched reverse genetics for the pestivirus Bungowannah virus (BuPV), where cDNA flanked by a hammerhead ribozyme sequence at the 5′ end and the hepatitis delta ribozyme at the 3′ end was placed under the control of the CMV RNA polymerase II promoter. Infectious recombinant BuPV could be rescued from pBuPV-DNA-transfected SK-6 cells and it had very similar growth characteristics to BuPV generated by conventional RNA-based reverse genetics and wild type BuPV. Subsequently, DNA-based E^RNS^ deleted BuPV split genomes (pBuPV∆E^RNS^/E^RNS^)—co-expressing the E^RNS^ protein from a separate synthetic CAG promoter—were constructed and characterized in vitro. Overall, DNA-launched BuPV genomes enable a rapid and cost-effective generation of recombinant BuPV and virus mutants, however, the protein expression efficiency of the DNA-launched systems after transfection is very low and needs further optimization in the future to allow the use e.g., as vaccine platform.

## 1. Introduction

Bungowannah virus (BuPV) is an atypical pestivirus (species *Pestivirus F*) within the genus *Pestivirus* of the *Flavivirdae* family [1]. The virus was isolated for the first time in 2003 from a large Australian integrated pig farm during an outbreak of sudden death in young pigs, followed by an increase in stillbirth [2,3]. Although BuPV represents a potential threat to commercial pig farming, it has not yet been reported from any other region or country [4,5].

BuPV has a positive sense RNA genome that is approximately 12.6 kb in length. A single open reading frame, flanked by 5′ and 3′ non-translated regions, encodes a polyprotein, which is co- and post-translationally processed into structural proteins (C, E^RNS^, E1, E2) and non-structural proteins (N^PRO^, p7, NS2/NS3 (NS2, NS3), NS4A, NS4B, NS5A, NS5B) [2]. The envelope protein E^RNS^ as well as the non-structural protein N^PRO^ are unique to pestiviruses.

Genomic and antigenic properties of BuPV, as well as its broad in vitro host cell tropism, indicate remarkable distance to previously described pestiviruses [6,7,8,9]. In general, pestiviruses infect host-specific cells of ruminant, porcine, or sheep origin. Classical swine fever virus (CSFV) can only infect porcine cells efficiently, while bovine viral diarrhea virus (BVDV) and border disease virus (BDV) have the potential to infect broader host spectra [10,11,12,13,14]. However, only BuPV could infect cell lines of African green monkey, bat, human, and mouse origin [9].

To study the special characteristics of BuPV in detail, a robust reverse genetics system (RGS) is essential. For other pestiviruses, such as CSFV or BVDV, many RGSs based on cDNA copies of the viral genome cloned into plasmid or bacterial artificial chromosome (BAC) vectors have already been reported [15,16,17,18,19,20]. Furthermore, recombinant pestiviruses were generated by full-length genome RT-PCR-based amplification and direct RNA generation from the amplicons without cloning steps [21]. All these techniques have in common the use of a bacteriophage T7 or SP6 RNA polymerase promoter for in vitro transcription to synthesize infectious positive strand RNA, which is subsequently transfected into cells to produce infectious virus progeny.

Here, we report a first dual promoter DNA-launched BuPV RGS, which is based on a cDNA plasmid (pBuPV), with a cytomegalovirus (CMV) immediate-early promoter as well as the bacteriophage RNA polymerase T7 (T7) promoter upstream of the BuPV genome in a mammalian expression vector. For the generation of correct 5′ and 3′ ends, self-cleaving ribozyme sequences were inserted. This construct enables the transcription of the BuPV DNA by the CMV promoter in the nucleus and by the T7 promoter in the cytoplasm of polymerase expressing BSR cells (BSR-T7/5), where the latter serves as proof of principle to demonstrate that the modifications of the genome do not affect the transcription. This plasmid allows the rescue of infectious virus without in vitro RNA synthesis, and the virus can be passaged efficiently. We also established a split genome construct (pBuPV∆E^RNS^/E^RNS^) with a large deletion in the *E^RNS^* gene, preventing the efficient generation of virus progeny, and a synthetic CAG promoter followed by the genomic region encoding the BuPV-E^RNS^ protein downstream the T7 termination signal (T7_term_) for the expression of BuPV-E^RNS^ and the production of single-round infectious particles (SRIPs) via *trans*-complementation. The particles generated in this way should be able to pass through an additional replication cycle to infect surrounding cells, but are not expected to be capable of further propagation.

## 2. Materials and Methods

### 2.1. Cells and Viruses

SK-6 cells (RIE262, Collection of Cell Lines in Veterinary Medicine (CCLV), Friedrich-Loeffler-Institut, Insel Riems, Germany) and BSR-T7/5 cells, constitutively expressing the T7 polymerase (RIE583, CCLV) [22], were grown in Dulbecco’s Modified Eagle Medium (DMEM) supplemented with 10% fetal calf serum (FCS) at 37 °C and 5% CO_2_. rBuPV^RNA^ was generated after RNA transfection of SK-6 cells with in vitro transcribed RNA from the previously described synthetic cDNA clone pA/BV [9]. The virus was propagated in SK-6 cells and virus stocks were generated after three cell culture passages.

### 2.2. Plasmid Construction

All plasmids were prepared by standard molecular biological methods and plasmid DNA was purified by the Qiagen Plasmid Midi kit (Qiagen, Hilden, Germany). The identity of the constructs was confirmed by Sanger sequencing using the Big Dye^®^ Terminator v1.1 Cycle sequencing kit (Applied Biosystems, Foster City, CA, USA) and appropriate primers. Nucleotide sequences were read with an automatic sequencer (3130 Genetic Analyzer; Applied Biosystems, Foster City, CA, USA) and analyzed using Geneious software (version 10.2.3.). Primers used for cloning procedures are shown in Table 1.

The infectious clone pBuPV, the basis for DNA-launched BuPV, was constructed by multiple cloning steps. In a first step, donor splice sites, that were detected in the BuPV genome with a confidence >0.65 using the NetGene2 Server (http://www.cbs.dtu.dk/services/NetGene2 [23,24]; Table S1), were mutated in the full-length clone “pA/BV.” For this, fusion PCR was applied, which is a restriction-free cloning method [7], using the Phusion^®^ High-Fidelity PCR kit (New England Biolabs, Ipswich, MA, USA) and the primers Ph_Donor_C-Erns_F, Ph_Donor_Erns_R, Ph_Donor_Erns_F, Bungo_2534R, Bungo_Npro_Mut_Donor_F, Bungo_1079R, Bungo_Donor_IV_F, Bungo_Donor_IV_R_new, Bungo_Donor_V_F, Bungo_6186_R, Bungo_Donor_VI_F, Bungo_Donor_VI_R_new, Bungo_Donor_VII_F, Bungo_Donor_VII_R, Bungo_Donor_VIII_F, Bungo_Donor_VIII_R, Bungo_Donor_IX_F, and Bungo_Donor_IX_R (biomers.net GmbH, Ulm Germany), resulting in plasmid “pBV_opt.”

For pBuPV generation, the well characterized plasmid pHaHd was used, which contains in addition to the CMV and T7 promoters, ribozyme sequences for the generation of correct genome sequences. In order to insert the optimized BuPV-specific cDNA into plasmid pHaHd [25] by linear-to-linear homologous recombination (LLHR) [26], plasmid pHaHd was linearized with *BglII* and used as template for PCR amplification of a linear vector fragment with the Phusion^®^ High-Fidelity PCR kit and primers pHaHd_F and pHaHd_R. A full-length BuPV-specific PCR fragment was amplified by using plasmid DNA pBV_opt as template and primers Bungo_LLHR_F and Bungo_LLHR_R. To allow homologous recombination of the virus- and vector-specific fragments, 50 nucleotide-long vector specific homology arms were included in the primer sequences. Furthermore, for correct cleavage by the synthetic hammerhead ribozyme (HHr), five nucleotides complementary to the 5′ end of the BuPV-genome had to be inserted into primer Bungo_LLHR_F (Table 1). Thereafter, the PCR fragments were digested with *DpnI* to remove residual template DNA and gel purified (QIAquick Gel Extraction kit; Qiagen, Hilden, Germany). Subsequently, both PCR fragments were subjected to LLHR [26]. Recombination was performed by electroporation of both DNA fragments in the *E. coli* strain GB05-dir (Gene Bridges, Heidelberg, Germany). In brief, fresh overnight cultures in lysogeny broth (LB) medium were incubated at 37 °C for 1.5 h and RecE/RecT recombination was induced by L-Arabinose. After an additional incubation period at 37 °C for 30 min, the cells were washed two times with ice-cold water. A total of 100 ng of the amplified DNA fragments were added to the pelleted bacteria and electroporation was done at 1350 V, 50 µF, and 600 Ω by using the Gene pulser Xcell Electroporation System (Bio-Rad, Hercules, CA, USA).

Plasmid pBuPV∆E^RNS^ with a deletion of 448 bases within the E^RNS^ protein (aa 328–483) was generated by fusion PCR using pBuPV as DNA template and primer pair Bungo_dERNS_F and Bungo_2164R. For construction of the split genome plasmid pBuPV∆E^RNS^/E^RNS^, plasmid pCAGGS_BuPV-E^RNS^ [27] was digested with *SmaI* and *NotI* and the E^RNS^ comprising fragment was ligated into plasmid pBuPV∆E^RNS^, digested with *PmeI* and *NotI*. Further details of the plasmid constructions are available on request.

### 2.3. cDNA Stability

The plasmid pBuPV was propagated for 10 passages in *E. coli*. Subsequently, the DNA was purified using the Qiagen Plasmid Mini kit (Qiagen, Hilden, Germany) and analyzed by *HindIII* digestion. DNA preparations of passages 5 and 10 were also used to transfect SK-6 or BSR-T7/5 cells and investigate for virus rescue.

### 2.4. Transfection and Virus Rescue

DNA transfections of plasmids pBuPV, pBuPV∆E^RNS^ and pBuPV∆E^RNS^/E^RNS^ (2 µg DNA each) into SK-6 (plasmid pBuPV) or BSR-T7/5 (all plasmids) cells were performed by using Lipofectamine™ 2000 Transfection Reagent (Invitrogen, Carlsbad, CA, USA) according to the manufacturer’s protocol. The transfected cells were seeded and incubated for three days at 37 °C and 5% CO_2_. For recovery of infectious viruses, supernatants of the transfected cells were harvested three days post transfection (p.t.) and passaged on SK-6 cells. At the day of collection, replication of BuPV was monitored by immunofluorescence (IF) staining using monoclonal antibodies. Virus stocks of pBuPV were prepared after four cell culture passages. The identity of the recombinant viruses was confirmed by RT-PCR and sequence analysis using appropriate primers. For RT-PCR, total RNA of virus-infected cells was extracted using the QIAamp Viral RNA Mini kit (Qiagen, Hilden, Germany) according to manufacturer’s instructions, and the cDNA was amplified using the OneStep RT-PCR kit (Qiagen, Hilden, Germany).

### 2.5. Immunofluorescence Assay

Transfected or infected cells were fixed and permeabilized with 80% acetone on ice for 15 min. After 30 to 45 min incubation with monoclonal antibodies specific for BuPV-E^RNS^ (682/43C3, diluted 1:20; M. Dauber, Friedrich-Loeffler-Institut, Insel Riems, Germany), for BuPV-E2 (682/45F12, diluted 1:20; M. Dauber, Friedrich-Loeffler-Institut, Insel Riems, Germany) or the pan-*Pestivirus* NS3 antibody WB112 (diluted 1:500; CVL, Weybridge, UK), the cells were washed twice with phosphate buffered saline (PBS). Thereafter, cell cultures were incubated with a goat anti-mouse Ig Alexa-488 conjugate (1:1000; Thermo Fischer scientific Inc., Waltham, MA, USA) for 30 min and analyzed by using a fluorescence microscope (Nikon Eclipse; Nikon GmbH, Düsseldorf, Germany).

### 2.6. Virus Titration and Growth Kinetics

SK-6 cells were infected with recombinant BuPV recovered from pBuPV (rBuPV^DNA^) and recombinant BuPV recovered from pA/BV (rBuPV^RNA^) at a multiplicity of infection (M.O.I.) of 1. Supernatants were collected at 0, 8, 24, 48, and 72 h post infection (p.i.), and virus titers were calculated as a 50% tissue culture infective dose per ml (TCID_50_/_mL_) after IF staining.

## 3. Results and Discussion

Reverse genetics systems are important tools that enable the investigation of viral genes, viral replication cycles, or pathogenesis, and allow for the development of safe and efficacious vaccines. Infectious virus production from DNA plasmid transfections into mammalian cells using RNA polymerase I (pol I) or RNA pol II systems had been described for several RNA viruses [28,29,30,31,32]. These systems allow a simple and stable virus rescue, faster and less costly than conventional RNA-based reverse genetics. RGS using a pol I-promoter [33] or a pol II-promoter [34] were also described previously for the pestivirus CSFV. Both systems allowed the rescue of infectious CSFV with high virus titers. In our study, we established a dual promoter BuPV infectious clone “pBuPV” with both the CMV pol II promoter and the bacteriophage T7 promoter, which allows virus generation via DNA through the nucleus (CMV pol II promoter) or cytoplasmatic T7-based generation. The T7-based generation served as a proof of principle to demonstrate that the modifications of the genome do not affect the transcription.

**Figure 1:**
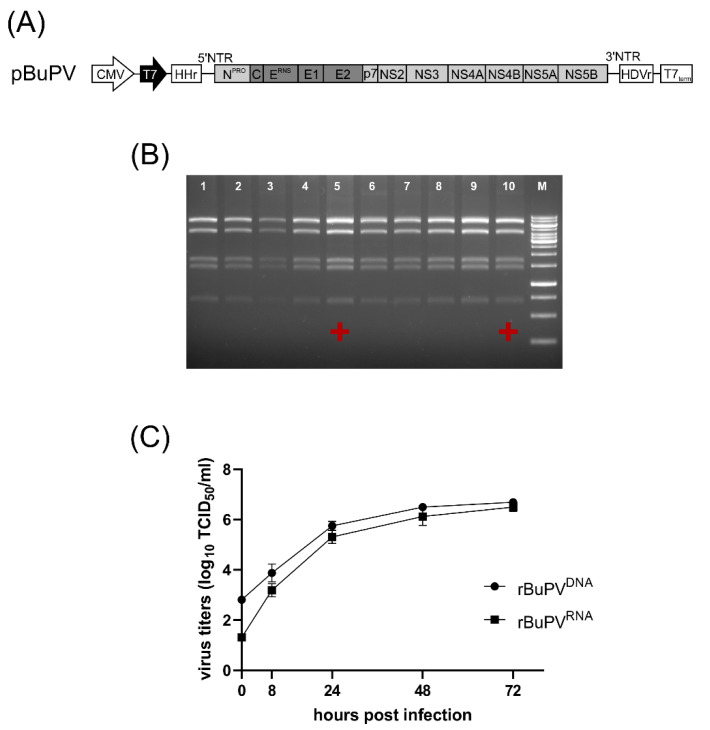
Plasmid pBuPV, genetic stability and rescue of recombinant rBuPV^DNA^. (**A**) Schematic representation of the RNA Polymerase II-based plasmid pBuPV encoding full-length BuPV cDNA. Indicated are cytomegalovirus immediate-early (CMV) RNA Polymerase II promoter (open arrow), bacteriophage RNA polymerase T7 (T7) promoter (shaded arrow), hammerhead ribozyme (HHr), 3′ hepatitis delta virus ribozyme (HDVr), and T7 terminator sequence (T7_term_). (**B**) Stability of the full-length cDNA clone pBuPV. The primary plasmid (P0) was passaged 10 times in *E. coli* DH10B (P1–P10) and investigated by restriction analysis using *Hind*III. **+** indicates generation of infectious rBuPV^DNA^ in rescue experiments. (**C**) Multi-step growth curves determined after infection of SK-6 cells with rBuPV^DNA^ (rescued by DNA transfection of pBuPV) or rBuPV^RNA^ (rescued by transfection of in vitro transcribed RNA of the infectious cDNA clone pA/BV) at an M.O.I. of 1 showed similar growth characteristics for both viruses.

In order to prevent the viral RNA from being spliced in the nucleus, donor splice sites with confidence >0.65 detected in N^PRO^, C, E^RNS^, E2, p7, and the other non-structural proteins were mutated in pA/BV (Table S1). Subsequently, the optimized BuPV-specific cDNA was used for construction resulting in plasmid pBuPV. In this construct, downstream of the CMV and bacteriophage T7 promoters, the BuPV genome termini were flanked by sequences coding for a synthetic HHr and hepatitis delta virus ribozyme (HDVr) to generate precise 5′- and 3′-terminal sequences. Downstream of the HDVr sequence, a T7 termination (T7_term_) signal allows transcription termination (Figure 1A).

The stability of plasmid pBuPV in bacteria was investigated by 10 serial cloning and passaging cycles in *E. coli* DH10B cells. Restriction enzyme analysis using *Hin*dIII provided some indication about the genetic stability of the construct, and the same restriction pattern for P0 (primary construct) and passages P1 to P10 were observed (Figure 1B). In transfection experiments, the DNA-launched recombinant rBuPV^DNA^ was analyzed for RNA replication, expression of BuPV proteins, and virus growth in both SK-6 and BSR-T7/5 cells. At 72 h p.t., CMV-driven expression of the BuPV proteins NS3 (anti-NS3), E2 (anti-E2), and E^RNS^ (anti-E^RNS^) was detected by IF staining of the pBuPV-transfected SK-6 cells.

**Figure 2:**
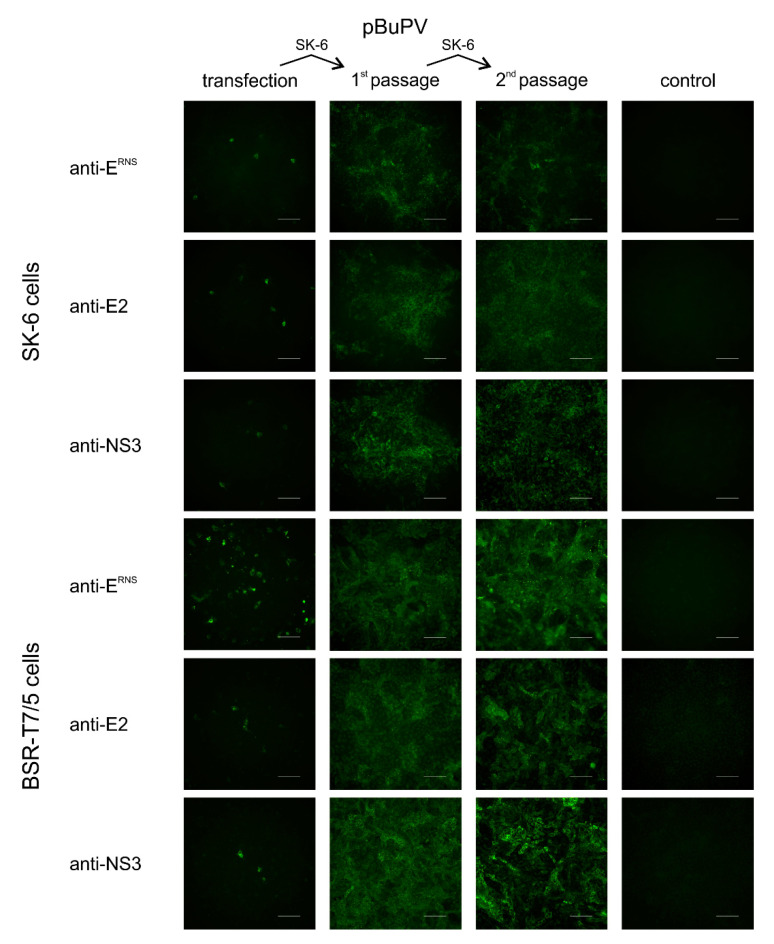
Rescue of rBuPV^DNA^ in BSR-T7/5 and SK-6 cells. Cells were transfected with plasmid pBuPV. At 72 h p.t, IF staining with pan-pesti NS3-specific mab WB112 (anti-NS3), and E2-specific and E^RNS^-specific mabs verified expression of NS3, E2 and E^RNS^ in transfected cells. At this time, recombinant virus in the supernatants was transferred to SK-6 cells (1st passage), and later on transferred for a 2nd passage. E2, E^RNS^ and NS3-positive cells indicated the generation of infectious progeny virus at 72 h p.i. in both transfected SK-6 and BSR-T7/5 cells. Scale bars indicate 100 µm.

Bacteriophage T7 RNA polymerase (T7-RNA-Polymerase)-driven cytoplasmatic expression of all proteins could be observed as well after transfection in BSR-T7/5 cells; DNA-transfection of SK-6 cells and transfection in BSR-T7/5 cells resulted in single NS3, E2, and E^RNS^ expressing cells (Figure 2). Cell culture supernatants collected from both transfected cell lines were inoculated into fresh SK-6 cells and the presence of recombinant BuPV (rBuPV^DNA^) particles in supernatants of both cell lines could be confirmed by IF analysis. The rescued rBuPV^DNA^ could be efficiently passaged in SK-6 cells (Figure 2). Virus rescue was possible, regardless of the bacterial passage number of pBuPV in *E. coli*, indicating once more the general stability of the plasmid (data not shown). Virus stocks produced from pBuPV-transfected SK-6 cells after four passages in SK-6 cells with a titer of 10^6.25^ TCID_50_/_mL_ were used for growth kinetics analyses in comparison to rBuPV generated by the previously established T7-RNA-Polymerase based RGS [9]. Analysis of the multi-step growth curves in SK-6 cells revealed similar growth characteristics for both viruses, and final virus titers of 10^6.6^ TCID_50_/_mL_ (rBuPV^DNA^) and 10^6.5^ TCID_50_/_mL_ (rBuPV^RNA^) could be determined at 72 h p.i. (Figure 1C).

In a next step, we were interested in the production of BuPV single-round infectious particles (SRIPs), since RNA-based SRIPs generating systems have already been described for several other flaviviruses [35,36,37,38]. SRIP production relies on the transfection of in vitro transcribed replicon RNA with deletions within the genomic region encoding for one of the structural proteins C, E^RNS^, E1, or E2 in cells stably expressing either the protein missing in the replicon or all structural proteins [39,40,41,42,43]. The packaged replicon particles are infectious, but progeny virus cannot spread from the infected cells, because the packaged replicon genome lacks the respective structural protein genes. In experimental animal studies, packaged replicon particles were proven to be appropriate for the development of non-transmissible, life attenuated pestivirus marker vaccine candidates [44,45,46]. However, the production of the replicon particles is time-consuming and needs the establishment of *trans*-complementing cell lines, which in many cases do not allow further passaging of the packaged pestivirus replicon particles.

**Figure 3:**
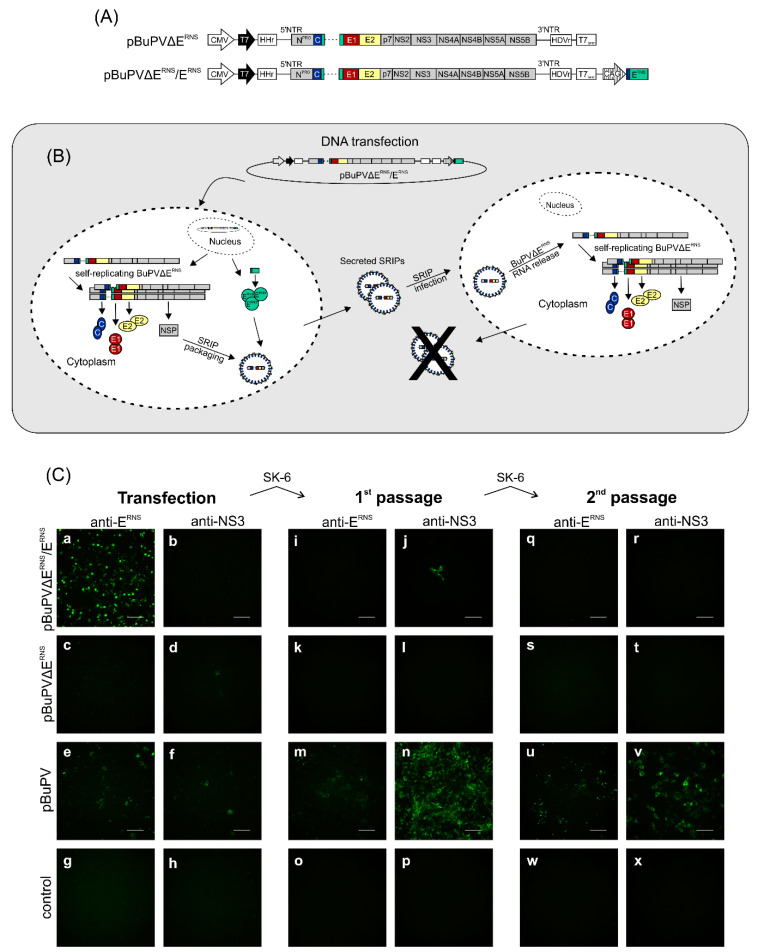
Schematic representation of the plasmids pBuPV∆E^RNS^ and pBuPV∆E^RNS^/E^RNS^ and production of rBuPV and BuPV-SRIPs. (**A**) The replicon construct pBuPV∆E^RNS^ was generated on the basis of the CMV immediate early promoter containing plasmid pBuPV by partial deletion of the E^RNS^ encoding genomic region (aa 328–483); the split genome plasmid pBuPV∆E^RNS^/E^RNS^ contains two eukaryotic promoters. The CMV promoter (open arrow) controls transcription of BuPV replicon RNA, BuPV∆E^RNS^, which expresses the non-structural protein genes and the structural protein genes C, E1, and E2. The CAG promoter (shaded arrow) downstream T7_term_ directs the expression of BuPV-E^RNS^. Indicated is also the T7 promoter (black arrow), and the hammerhead ribozyme (HHr), and the 3′ hepatitis delta virus ribozyme (HDVr), which are important for the generation of the correct termini of the transcribed replicon RNAs. (**B**) Generation and operation mode of BuPV single round infectious particles (SRIPs) [47]. When cDNA of pBuPV∆E^RNS^/E^RNS^ is transfected into susceptible cells, RNA-transcription starts in the nucleus under the control of the CMV promoter. The structural proteins C, E1, E2, and the non-structural proteins (NSP) are then expressed in the cytoplasm, while E^RNS^ is expressed by the CAG promoter. The self-replicating, truncated RNAs can be packaged in SRIPs by the four essential structural proteins. The secreted SRIPs are able to infect new cells. The released RNA replicates autonomously in the cytoplasm and allows the expression of the structural proteins C, E1, and E2 but not of E^RNS^. Therefore, no further SRIPs can be produced and spread again (self-restriction). (**C**) IF analysis of BSR-T7/5 cells transfected with pBuPV∆E^RNS^, pBuPV∆E^RNS^/E^RNS^ or pBuPV (a–f) or SK-6 cells infected with supernatants of DNA-transfected cells (1st passage, i–n) or infected with supernatants collected from cells after the first infection (2nd passage, q–v). IF staining using anti-NS3 or anti-E^RNS^ monoclonal antibodies was performed at 72 h p.t. and 72 h p.i., respectively. Non-transfected or uninfected cells were used as control (g–h, o–p, and w–x). Scale bars indicate 100 µm.

Here, DNA-based SRIPs were produced as packaged BuPV replicon particles. Other DNA-based flavivirus SRIPs generating systems are mostly based on co-transfection of two expressing plasmids directly in eukaryotic cells, a subgenomic replicon plasmid, which lacks the structural protein-coding region, and a structural protein-expressing plasmid [35,36,37,38]. In addition, split genomes with two CMV promoters in back-to-back orientation, directing either the transcription of a capsid-deleted replicon RNA or the transcription of capsid-encoding mRNA had been described [47]. Since this strategy was described for the flavivirus West Nile virus, but had not been applied to pestiviruses up to now, we first constructed pBuPVΔE^RNS^, which is a DNA-based replicon plasmid with a deletion of a large portion (codons 328–483) of the genome region encoding E^RNS^ (Figure 3A, upper panel). Subsequently, this plasmid was used for the establishment of the split genome construct pBuPVΔE^RNS^/E^RNS^ (Figure 3A, lower panel). In transfection experiments using pBuPVΔE^RNS^ and BSR-T7/5 cells, transient expression of NS3 could be detected at 24 h p.t. by IF staining, while expression of BuPV-E^RNS^ could not be observed (Figure 3C, panels c–d). No infectious recombinant BuPV could be recovered, even after serial passages in SK-6 cells (Figure 3C, panels k–l and s–t).

By insertion of a synthetic CAG promoter and the genomic region encoding the N-terminal signal sequence and the BuPV-E^RNS^ protein in plasmid pBuPVΔE^RNS^ downstream the T7_term_ signal, the split genome plasmid pBuPVΔE^RNS^/E^RNS^ was generated (Figure 3A, lower panel). This construct is capable of transcribing two separate RNA species from two different promoters.

The CMV promoter directs the transcription of BuPV replicon RNA BuPVΔE^RNS^, which expresses all non-structural protein genes and the structural protein genes *C*, *E1*, *E2*, and a truncated *E^RNS^* gene (ΔE^RNS^), whereas a synthetic CAG promoter [48] downstream of the BuPV replicon genome directs transcription of mRNA encoding full-length E^RNS^ for complementation. Together, the two promoters allow the expression of the complete BuPV genome including all structural proteins. The E^RNS^-deleted replicon genome is amplified by the BuPV non-structural proteins NS3, NS4A, NS4B, NS5A, and NS5B and can be packaged by the structural proteins C, E^RNS^, E1, and E2, essential for virus assembly, to generate SRIPs (Figure 3B). Secreted SRIPs are able to infect surrounding cells, where the replicon RNA can be replicated. As the structural proteins C, E1, and E2, but not BuPV-E^RNS^ can be expressed from this RNA by the non-structural proteins, the RNA cannot be packaged again into new particles and no further spread of the SRIPs is possible (Figure 3B) resulting in a self-restricted system.

To examine the ability of the split genome plasmid pBuPVΔE^RNS^/E^RNS^ to produce SRIPs, BSR-T7/5 cells were transfected with pBuPVΔE^RNS^/E^RNS^ and compared with cells transfected with the replicon plasmid pBuPVΔE^RNS^ and the full-length plasmid pBuPV. Autonomous replication of the newly synthesized BuPV RNA was shown by the expression of BuPV-NS3 in cells transfected with plasmids pBuPVΔE^RNS^ and full-length pBuPV. However, unexpectedly no NS3 expression was observed for the SRIPs, which might be due to the low transfection efficiency, especially since also only single positive cells could be shown for the other constructs. Expression of E^RNS^ could only be detected in cells transfected with pBuPVΔE^RNS^/E^RNS^ and the full-length pBuPV, but not in pBuPVΔE^RNS^ DNA transfected SK-6 cells (Figure 3C, panels a, c, and e). The observation that the SRIPs showed an increased E^RNS^ expression (Figure 3C, panel a) could be related to the fact that the inserted sequence was optimized for the expression system applied in this study.

When in vitro-transcribed RNA produced from an infectious cDNA clone of BuPV was transfected, the deletion of E^RNS^ still allowed the generation of infectious particles [27]. However, in this study there were no indications of cell-to-cell spread in pBuPVΔE^RNS^ DNA-transfected cells or production of SRIPs in transfection supernatants (Figure 3C, panels c, d, k, and l), while transfection with pBuPV resulted in single NS3 and E^RNS^ positive cells. In cells transfected with pBuPVΔE^RNS^/E^RNS^, amplified BuPVΔE^RNS^ replicon RNA was packaged into BuPV-SRIPs (Figure 3C, panel a). The infectivity of BuPV-SRIPs and rBuPV was also demonstrated after inoculation of the supernatants of the pBuPVΔE^RNS^/E^RNS^ DNA-transfected cells to fresh SK-6 cells (1st passage) as indicated by the IF-detection of NS3 at 72 h p.i. Here, replicon RNA amplified itself since no additional SRIPs were produced; only single cells infected with BuPV-SRIPs were observed by IF staining using the anti-NS3 mab and no positive signals could be detected by E^RNS^ staining (Figure 3C, panels i and j).

In contrast, after one passage, the cells infected with rBuPV produced large foci of BuPV-NS3 and -E^RNS^ protein-positive cells resulting from virus replication and spread (Figure 3C, panel m and n). The supernatants from all investigated clones obtained after infection were again transferred to fresh cells (2nd passage), but infectious progeny virus was only detected in rBuPV infected cells (Figure 3C, panels u, v).

While the newly established DNA-based system is well suited to produce infectious viruses, it unfortunately shows only a very low transfection efficiency, as demonstrated by the IF staining of relatively few positive cells at 72 h after transfection (Figure 2 and Figure 3). A possible explanation for the reduced efficiency might be the still insufficient removal of donor splice sites. As BuPV does not naturally replicate in the cell nucleus, we have modified several donor splice sites, although additional splice sites may have an impact on efficiency [49,50]. In addition, further optimization attempts such as the modification of the CMV promoter or the insertion of a polyA tail were made following other flavivirus systems, but they did not lead to an increase in efficiency after transfection for the newly established BuPV system (data not shown).

However, also SRIP systems for the flavivirus West Nile virus showed reduced efficiency after transfection, but still were able to induce neutralizing antibodies and confer protection in immunized mice and horses [47]. Whether this is the case for the BuPV system as well, needs to be evaluated in vaccination experiments. Nevertheless, since a high transfection efficiency might be important for SRIP systems, further optimization of the BuPV system will be necessary and useful. Roby et al. could increase the SRIP production of their beta-galactosidase expression system by using an elongations factor EF1α promoter for the expression and optimized the codon of the capsid protein [51]. Thus, the replacement of the CMV promoter by another, more efficient promoter in SK-6 cells, e.g., the CAG promoter, might be an option to improve the BuPV system in the future. In addition, it could be explored whether other structures of the system, such as the ribozyme sequences, influence the efficiency as was shown previously [31,52,53].

## 4. Conclusions

In summary, we established a cDNA clone for the rescue of infectious BuPV using an RNA polymerase II-driven system. The full-length clone pBuPV enables further investigation of the atypical pestivirus BuPV by rapid and cost-effective generation of BuPV mutants. Even though the split-genome strategy has to be optimized due to its very low transfection efficiency in cell culture, it could allow the establishment of a platform for *trans*-complementation of viral proteins with a single plasmid. A more efficient version could be an attractive alternative to conventional complementation approaches and will therefore be a major focus for the future research in this field.

## Figures and Tables

**Table 1:** Primer sequences for plasmid construction.

Construct	Primer	Sequence 5′-3′
pBV_opt	Ph_Donor_C-Erns_F	GCCTGCCTATTGGTCGTGCCCGTGGGCTCCACCAACGTGACACAATG
	Ph_Donor_Erns_R	TCACCCCTAAGTCTGCATCGTATCTGCATGTGACTGCGCACTC
	Ph_Donor_Erns_F	GTTGACGGTTACACCGAGGTGGTGGAGAAGGCCAGGTCAAGTGG
	Bungo_2534R	CGCTAATGCGTACATGAATTC
	Bungo_Npro_Mut_Donor_F	CTTTGTACAAACCAAGAGAGATGTGAGGGATCCAAGTGTGTA
	Bungo_1079R	GTGGCATCTGGTGGTCTAG
	Bungo_Donor_IV_F	GGCACTTGTATTGACAAAGAGGGTAGCGTGCAATGCTACATAGGGGA
	Bungo_Donor_IV_R_new	TCTTTAGTTCCCTCTTCGGCGCGTACTAAACCGACGAAGTAGACCAC
	Bungo_Donor_V_F	GCCTACACACACCCTGGAGGTGTAAGCAGTGTGATGCATGTCACCGC
	Bungo_6186_R	CACCGAACCTATGTATTTTTGACATCACTGCCAACTGTTC
	Bungo_Donor_VI_F	ATCACCAAATCCAACAAATTCTCGAGGGTGGGAAAGAATATGTCGGCCAAGCCTA
	Bungo_Donor_VI_R_new	GGACCCCCCATAGACCGTATTTCTTGATGTCACCGGCATGCTCTTGCAAGTATTC
	Bungo_Donor_VII_F	GGCCAGAAAAATTGCCAGTAGTAAGGGCCCAGACCAGTACCAAAG
	Bungo_Donor_VII_R	CTGGTTGACCACTTCCCCTTTGTCCTTCTCTTATGTAGACGTTTC
	Bungo_Donor_VIII_F	GTAGATGATTGGATGGAAGGAGATTATGTAGAAGAAAAAAGACC
	Bungo_Donor_VIII_R	GGCCCCTTGATCGCAAAGGCTTCGCCAAAACTTTTCTCAGTTATC
	Bungo_Donor_IX_F	GGTCAACCAGACACTAGCGCTGGAAATAGTATGTTGAATGTACT
	Bungo_Donor_IX_R	GACAAGCAGGCATATTCTTCGTACGAGGGGGTTCCAAGAATAC
pBuPV	Bungo_LLHR_F	CGTCGTTATACCTGATGAGTCCGTGAGGACGAAACCCGGAGTCCCGGGTCGTATAACGACAGTAGTTCAA
	Bungo_LLHR_R	TTCGGATGCCCAGGTCGGACCGCGAGGAGGTGGAGATGCCATGCCGACCCAGGGCTTTTTGGAACTGTGC
	pHaHd_F	TGGGTCGGCATGGCATCTCC
	pHaHd_R	GACCCGGGACTCCGGGTTTCGTCCTCACGGACTCATCAGGTATAACGACGACTAGCCAGCTTG
pBuPV∆E^RNS^	Bungo_dERNS_F	CATCTAGCAGCAGACTATGAAAGTAAGATTGAAAACACCAAGA
	Bungo_2164R	CATCACGAAGTCCCTGTTGTC

## Supplementary Materials

Supplementary materials can be found at https://www.mdpi.com/1999-4915/12/8/847/s1. Table S1: Mutated sequences for donor splicing site deletion.
