# Supplementary material for: Single-Round Infectious Particle Production by DNA-Launched Infectious Clones of Bungowannah Pestivirus
Source: Viruses. 2020 Aug 4;12(8):847. doi: 10.3390/v12080847 (PMC7472241; doi:10.3390/v12080847)
Supplement: Supplementary file 1 [file viruses-12-00847-s001.pdf]

Table S1. Mutated sequences for donor splicing site deletion.

Protein	Confidence	Sequence	New sequence
N^{pro}	0.68	ACCAAGAGAG^GTGCGAGGGA	ACCAAGAGAG^**A**TG**T**GAGGGA
Capsid	0.91	GGTCGTGCCG^GTAGGGTCCA	GGTCGTGCC**C**^GT**G**GG**C**TCCA
E^{rns}	0.82	TCACATGCAG^GTATGATGCA	TCACATGCAG^**A**TA**C**GATGCA
	0.82	TTACACCGAG^GTAGTAGAGA	TTACACCGAG^GT**G**GT**G**GAGA
E2	0.85	GACAAAGAAG^GTAGTGTGCA	GACAAAGA**G**G^GTAG**C**GTGCA
p7	0.90	CGTCGGTTTG^GTGAGGGCCG	CGTCGGTTT**A**^GT**AC**G**C**GCCG
NS3	0.83	CCCTGGAGGG^GTAAGTAGTG	CCCTGGAGG**T**^GTAAG**C**AGTG
NS4B	0.77	ATTCTCGAAG^GTGGGAAGGA	ATTCTCGA**G**G^GTGGGAA**A**GA
	0.70	GAGCATGCAG^GTGACATAAA	GAGCATGC**C**G^GTGA**T**ATAAA
NS5B	0.83	ATTGCCAGTG^GTAAGAGCCC	ATTGCCAGT**A**^GTAAG**G**GCCC
	0.70	TGGATGGAAG^GTGACTATGT	TGGAAGG**A**GA^**T**TATGTAGAA
	0.88	ATAAGAGAAG^GTCAGAGGGG	ATAAGAGAAG^G**A**CA**A**AGGGG
	0.83	ACTAGCGCAG^GTAATAGTAT	ACTAGCGC**T**G^G**A**AATAGTAT
	0.79	AAAAGTTTTG^GTGAGGCCTT	AAAAGTTTTG^G**C**GA**A**GCCTT
	0.83	GAACCCCCTG^GTAAGAAGAA	GAACCCCCT**C**^GTA**C**GAAGAA

Letters indicated in bold and underlined were changed to mutate the donor splice recognition sequences.
